# The mental health of officials who regularly examine child sexual abuse material: strategies for harm mitigation

**DOI:** 10.1186/s12888-023-05445-w

**Published:** 2023-12-13

**Authors:** Kimberly J. Mitchell, Ateret Gewirtz-Meydan, David Finkelhor, Jennifer E. O’Brien, Lisa M. Jones

**Affiliations:** 1 Crimes against Children Research Center, University of New Hampshire, Durham, NH 03824 USA; 2 School of Social Work, Faculty of Social Welfare & Health Sciences, University of Haifa, Haifa, Israel; 3 Department of Social Work, University of New Hampshire, Durham, New Hampshire 03824 USA

**Keywords:** Police wellness, Child sexual abuse material, Resiliency, Mental health

## Abstract

**Background:**

The current study aims to better understand the mental health and subjective well-being of investigators and forensic examiners exposed to child sexual abuse material (CSAM) by examining which components of this work are associated with elevated mental health conditions and decreased well-being, as well as the intra-personal and organizational variables that may mitigate harm and improve well-being.

**Methods:**

Police investigators, forensic examiners, and others connected with the criminal justice system from across the United States who were exposed to CSAM as part of their professions (N = 500) completed an anonymous online survey. Participants were recruited through connections with the National Criminal Justice Training Center.

**Results:**

Duration, frequency, amount, and content of CSAM exposure was not related to poorer mental health with the exception of exposure to violent CSAM which was related to elevated post-traumatic stress symptoms. Several agency-level practices and policies, such as the availability of an Officer Wellness Program and more frequently knowing the final case resolution, were related to better mental health and well-being. Harm mitigation strategies, such as talking to other officers investigating the case and taking breaks from the material being viewed, were also related to better mental health.

**Conclusions:**

Findings indicate that police agencies have options for implementing agency-level procedures and practices that have the potential to reduce the negative impact of CSAM investigations. Additionally, many investigators use strategies that are correlated with greater well-being, suggesting opportunities for improving training programs.

## Introduction

Previous researcher suggests that the prevalence of mental health conditions among law enforcement is higher than the general population [1, 2]. The most common mental health conditions among law enforcement are post-traumatic stress disorder (PTSD), depression, and anxiety [3]. A recent systematic review and meta-analysis suggested that 14.6% of police personnel suffer from depression,14.2% from PTSD, 9.6% a generalized anxiety disorder, and 8.5% report suicidal ideation [3].

Mental health conditions have a significant negative impact on one’s subjective well-being [4]. Subjective well-being is defined as the degree to which a person’s affective and cognitive appraisals of life are positive or negative [5–8]. While research on the mental health and subjective well-being of police officers [9] and mental health programs for police personnel [10] is growing, far less is known about the mental health and subjective well-being of investigators and forensic examiners who are regularly exposed to child sexual abuse material (CSAM). The current study sought to better understand the mental health and subjective well-being of investigators and forensic examiners exposed CSAM as part of their professions, examining which components of this work are associated with elevated mental health conditions and decreased well-being, as well as the intra-personal and organizational variables that may help mitigate harm and improve well-being.

## CSAM victimization

CSAM is a serious problem in the United States and worldwide, fostered by the growth of online and digital technologies [11, 12]. CSAM, also sometimes referred to as child sexual abuse images and child sexual exploitation materials, is defined as “any representation through publication, exhibition, cinematography, electronic means or any other means whatsoever, of a child, a person made to appear as a child or realistic material representing a child, engaged in real or simulated explicit sexual activity, or any representation of the sexual parts of a child for primarily sexual purposes” [13]. Since the expansion of the Internet in the mid-1990s, there has been a growing number of cases that involve the possession, distribution, and production of CSAM. The agency that receives international reports of CSAM from the technology industry and other sources reported 29 million images identified in 2021 [14]. Law enforcement personnel must carefully review these images for a variety of reasons, including to classify the criminal statutes that are being violated, to make decisions about further investigation and prosecution, and to confirm the identity of victims and suspects.

CSAM is varied and can include children engaged in sexual acts, depictions of children’s genitals and private parts for purposes of sexual arousal, violent acts of abuse, and involve very young children [15]. CSAM can take the form of still images, recorded videos, or live streaming video. Until 2010, CSAM was primarily produced by the abuser, but more recently, youth self-made images have contributed to these numbers [16]. Many of these youth-produced images are originally shared voluntarily with romantic partners, but later non-consensually posted and shared with others. Such images are nonetheless illegal if they depict people under the age of 18. Other youth-produced images are created at the request of the abuser [17].

## Impact of CSAM investigations

Given its unusual and often disturbing content, and the urgency to help protect children in dire circumstances, investigating CSAM can be extremely impactful to those who must review it [18, 19]. Previous research indicates that crimes involving child victims can be particularly distressing because investigators may have difficulty creating emotional distance between themselves and the material, identify with victims, be reminded of their own children as victims, or feel guilty that they or society have failed to protect victims [20, 21]. Varied effects of CSAM exposure for law enforcement personnel have been noted. Corrosive effects on investigators’ mental health [22, 23] can include secondary traumatic stress disorder (e.g., intrusive imagery, flashbacks, nightmares, and social withdrawal), increased generalized distrust of people, over protectiveness of children, and difficulty in relationships with partners and children [18, 24–27].

Alongside the extremely demanding work of CSAM investigations and the potential toll this work might have on investigators’ mental health, some studies suggest that, irrespective of the investigators’ gender or degree of exposure to CSAM, their subjective well-being was within the expected adult normative range [28, 29]. Such well-being may be explained by an array of personal coping strategies, organizational factors, and factors related to the investigation and CSAM itself.

Personal factors that are associated with better mental health include using humor and being able to set boundaries between one’s personal life and the job [25]. At the organizational level, positive supervisory support, agency support, psychological resources, proper training, and coworkers who can be relied upon had the strongest associations with better mental health and well-being [24, 25, 27, 30]. At the investigation level, having less of a sense of control over the case [25] and frequent exposure to disturbing materials was associated with feeling overwhelmed and higher levels of PTSD [30]. Investigators highlight the importance of reducing workload and having a comfortable physical environment (open office spaces, natural light, ventilation) [27]. The need for rigorous psychological and well-being support as well as building emotional awareness and resilience that could help prevent or manage the emergence of negative consequences generated by handling CSAM is also beneficial [22]. Interestingly, although CSAM contains disturbing content, police and child protection workers indicate that organizational factors (e.g., workplace climate) are more closely related to their stress level than the exposure to CSAM itself [31].

## The current study

Although these studies have made a significant contribution towards understanding the experience of CSAM investigators, gaps in the literature remain. Previous studies were conducted using qualitative methodologies [22, 25, 27], with a main goal of understanding investigators’ experiences. The current paper examines relationships between differing levels of CSAM exposure among forensic examiners and investigators and their mental health and subjective well-being using quantitative measures. Specifically, we examine the relationships between levels of CSAM (i.e., duration, frequency, quantity, content), departmental policies and practices, and personal characteristics (e.g., length of time in the field) and self-reported depression and anxiety symptoms, posttraumatic stress symptoms, and subjective well-being. Then, we examine different harm mitigation strategies used by examiners and investigators when working with CSAM to help reduce its impact.

## Methods

### Participants

Participants were 698 police investigators, forensic examiners, and others connected with the criminal justice system from the across the United States who conduct child sexual exploitation investigations. The current paper included participants who reported any CSAM exposure as part of their profession and had completed at least 85% of the survey questions, resulting in an analytic sample of 500 participants: 61.4% of participants were male; most were between the ages of 35–44 (39.8%) with an additional 21.6% aged 25–34 and 29.8% aged 45–54. The majority of participants reported their race as White (85.8%) and 7.3% were of Hispanic or Latino ethnicity. Further details of the sample are depicted in Table 1.

**Table 1:** Demographic, job, and agency characteristics

Characteristic	All (n = 500) % (n)
Gender	
Male	61.4 (307)
Female	37.4 (187)
Non-binary	0.2 (1)
Decline to answer	1.0 (5)
Age	
18–24	0.8 (4)
25–34	21.6 (108)
35–44	39.8 (199)
45–54	29.8 (149)
55–64	6.6 (33)
65–74	0.2 (1)
Decline to answer	1.2 (6)
Hispanic ethnicity	7.3 (35)
Race ^a^	
White	85.8 (429)
Black or African American	3.0 (15)
Asian or Pacific Islander	2.8 (14)
Native American or Alaska	1.2 (6)
Mixed racial background	3.0 (15)
Decline to answer	5.0 (25)
Marital status	
Married	70.2 (351)
Unmarried but living with partner	6.4 (32)
Separate or divorced	9.4 (47)
Widowed	0.8 (4)
Single (never married)	11.2 (56)
Decline to answer	2.0 (10)
Parent children under the age of 18 (any)	63.0 (315)
Grandchildren under the age of 18 (any)	9.6 (48)
Number of years in current position	
Less than 1 year	8.4 (42)
2–3 years	32.0 (160)
4–6 years	22.6 (113)
7–10 years	15.6 (78)
11–15 years	12.0 (60)
16–20 years	6.2 (31)
More than 20 years	3.2 (16)
Number of years in field	
Less than 1 year	1.2 (6)
2–3 years	6.6 (33)
4–6 years	11.2 (56)
7–10 years	16.8 (84)
11–15 years	22.0 (110)
16–20 years	19.6 (98)
More than 20 years	22.6 (113)
Work as part of the ICAC Task Force Program	87.8 (439)
Percentage of time work ICAC investigations	
Less than 25%	23.9 (105)
25 − 49%	22.5 (99)
50 − 74%	11.9 (52)
75% or more	41.0 (180)
Not sure	0.7 (3)
Place of residence	
Large city (population over 300,000)	21.8 (109)
Smaller city (population about 100,000-300,000)	27.8 (139)
Town (population about 20,000-100,000)	28.6 (143)
Small town (population about 2,500–20,000)	17.01 (85)
Rural area (population under 2,500)	4.0 (20)
Decline to answer	0.8 (4)
Types of crimes investigate ^a^	
Internet crimes against children	93.4 (467)
Other cybercrimes	43.8 (219)
Homicide	43.4 (217)
Fraud	37.4 (187)
Family and sexual violence	61.8 (309)
Crimes against property	33.2 (166)
Narcotics	25.4 (127)
Gang violence	14.4 (72)
Type of agency work for	
Federal	11.6 (58)
State	24.4 (122)
Local	62.6 (313)
Non-profit	1.0 (5)
Other	0.4 (2)

^a^ Multiple responses were possible

### Procedure

Participants were recruited through announcements at the July 2021 Virtual Conference of the Internet Crimes Against Children (ICAC) Task Forces and at the October 2021 ICAC Task Force Virtual Commanders Meeting, through the ICAC Task Force listserv, from trainings on investigations of internet crimes against children held by the National Criminal Justice Training Center (NCJTC), and through specific invitations to past NCJTC students with “forensic” in their title.

Participants completed an anonymous survey hosted through Qualtrics, an online survey data collection system. Participants were told the aim of the study was to understand the impact of work-related exposure to child sexual abuse material. The data collection period was July 2021 to December 2021. Participants were told that they could skip any questions they did not want to answer. To ensure full anonymity, all Qualtrics tracking features, like IP address, longitude, and latitude were turned off. Participants were also encouraged to take the survey while in “incognito” mode and were provided instructions on how to do this. The recruitment methodology using announcements at national conferences and trainings results in a convenience sample, in contrast to a probability sample; therefore, a meaningful response rate cannot be calculated. At the end of the survey, participants were provided with resources where they could learn more about trauma and well-being and to seek help if needed (e.g., National Suicide Prevention Lifeline, National Mental Health Information Center, the IACP mental wellness for police officers’ website). All data were collected under the approval of the University of New Hampshire Institutional Review Board.

### Measures

The measures consisted of a combination of established scales and those developed for the current study. Newly developed items were designed through interviews and consultations with criminal justice personnel and mental health providers.

#### Work Environment

*Department policies and practices* were measured using several items developed for the current study. Items queried the type of agency they worked for (e.g., state, local), number of full-time sworn officers, the existence of an Officer Wellness Program, the existence of other specialized training around wellness, other services available for well-being (e.g., peer counselors), and other accommodations to promote well-being (e.g., onsite gym, options to bring your dog to work). Participants were also asked to respond to 12 questions that asked how helpful they felt each was for helping them to stay healthy and productive in their work (e.g., longevity in law enforcement, having a strong family, having a hobby). Response options for these items consisted of a 4-point Likert scale ranging from 1 (not at all helpful) to 4 (extremely helpful). Participants were also given the opportunity to indicate if an item did not apply to them (e.g., being a parent).

#### CSAM Investigation Experience

Questions asked about department policies specific to CSAM, details of CSAM exposures, and CSAM harm mitigation strategies. All items were developed for the current study and described in more detail below.

*Department policies specific to CSAM.* Questions queried how long the participant had been working CSAM investigations, whether they were given preparation before first exposure, and whether they had any mental health testing or screening before they began work that involved viewing CSAM. Items also asked about attendance at a training program related to CSAM investigations. Participants were also provided with eight different policies or practices and asked whether each was available to personnel in their agency (e.g., staff meetings where reactions to work-related exposure to CSAM was discussed). Participants were asked whether they felt their agency offered ample vacation/personal time off, provided daily opportunities for CSAM investigators to debrief with other CSAM investigators, provided regular administrative updates about positive outcomes from CSAM investigations, and allowed them to tend to personal obligations during work hours using (non-paid) their own time. They were also asked to rate, in their agency, how much respect was given to personnel who investigate crimes involving CSAM (no or very little, some, a lot of respect) as well, on a scale of 1 (no problem at all) to 4 (a very big problem), how large of a problem they felt nine different issues were in their agency (e.g., not enough people to conduct forensic exams, volume of material/cases).

*CSAM exposures.* Questions about CSAM exposure identified how often (i.e., number of days per month) they reviewed CSAM, how much CSAM they viewed (i.e., number of still images, recorded videos, and live stream videos), and how often they reviewed 13 different types of content (e.g., children aged 5 or younger, involving sound) [never, sometimes, often, all the time]. They were asked how much control they felt they had over the work that was assigned to them (no control, some, a lot of control) and how often they hear about the final case resolution (never, sometimes, often, all of the time).

*CSAM harm mitigation strategies.* Twenty items asking about things participants did when reviewing CSAM to help them deal with the material they were viewing were developed for this study. Response options ranged from 1 (never) to 4 (often). Examples of items included: “taking break from the material I am reviewing”, “taking deep breaths”, “reducing the size of the image”, and “changing the image to black and white.”

#### Outcomes

*Depression and anxiety* were measured using the Patient Health Questionnaire-4 (PHQ-4) [32]. The scale presents a list of conditions, asking the participant to indicate how much each problem had bothered them in the past two weeks from 0 (not at all) to 3 (nearly every day). Items were combined to create a total scale score (*α* = 0.84) with higher scores representing more symptomatology.

*Posttraumatic stress symptoms (PTSS)* were measured using a shortened posttraumatic stress symptom checklist for DSM-5 (PCL-5; [33]) The scale presents four items that some people have in response to a very stressful experience (e.g., feeling distant or cut off from other people) and asks participants to indicate how much they have been bothered by each in the past month. Response options ranged from 1 (not at all) to 5 (extremely). Items were combined to create a total scale score (*α* = 0.79) with higher scores representing more posttraumatic stress symptomatology. In the current study it was important to know what type of stressful experience the participant was thinking about. As such, before being presented with the four conditions we asked participants to think of a stressful experience they had in the past month and indicate whether it was related to: (a) reviewing CSAM at work, (b) another type of case, (c) something else at work (not case specific), (d) something not related to work, and (e) had no stressful experience in the past month.

*Subjective well-being* was measured using a 7-item scale to assess satisfaction with different aspects of one’s life [34]. Items measured how much you think and feel your life is going well. Participants were asked to answer how true each of seven statements were about them on a 4-point scale ranging from 1 (not true about me at all) to 4 (mostly true about me). A sample item is “I have a lot to be proud of.” Reliability for the entire scale in the current study was excellent (α = 0.89). Items were summed to create a total scale score.

#### Participant demographics

Information gathered about the participants included their number of years in law enforcement, gender, age, race, ethnicity, marital status, number of children and/or grandchildren who are currently minors, and type of community (large city, small town, etc.).

## Results

### Mental health and well-being

Participants represented a range of levels of mental health symptoms and well-being. The mean score for depression and anxiety was 5.77 (SD = 2.31), ranging from a low of 3.25 to a high of 13.0. Variance was 5.33, skewness was 0.97, and kurtosis was 3.46 indicating a close-to-normal distribution (a normal distribution would have a skewness of 0 and a kurtosis of 3) with slightly more on the higher end. For PTSS, the mean value was 6.58 (SD = 2.78), ranging from a low of 3.25 to a high of 16.25. The variance was 7.72, skewness was 0.81, and kurtosis was 3.21 - similar to the mental health scale distribution. Finally, for subjective well-being, the mean value was 21.36 (SD = 3.44), ranging from a low of 9.43 to a high of 24.57. The variance was 11.87, skewness was − 1.11, and kurtosis was 3.67.

### Job details

Almost all (93.4%) participants worked cases that involved internet crimes against children (ICAC) (See Table 1). Investigating different types of crimes in addition to ICAC cases was common, with 61.8% working family and sexual violence crimes, 43.8% other cybercrimes, 43.4% homicide, 37.4% fraud, 33.2% crimes against property, 25.4% narcotics, and 14.4% gang violence. Most particpants worked as part of the ICAC Task Force Program (87.8%) and many worked for a local police agency (62.6%). Both years in the field and years in their current position varied from less than one year to more than 20 years.

### Bivariate relationships between characteristics of exposure to CSAM and mental health and well-being

Participants reported extensive exposure to CSAM in terms of duration, frequency, quantity, and content (See Table 2).

**Table 2:** Bivariate relationships between charactersitics of exposure to CSAM and mental health

	All (n = 500)	Depression/ anxiety	PTSS	Well-being
Characteristic	% (n)	β	β	β
Length of time working CSAM cases				
Less than 1 year	12.0 (60)	0.02	-0.01	0.03
2 to 3 years	33.2 (166)			
4 to 6 years	19.4 (97)			
7 to 10 years	18.6 (93)			
11 to 15 years	9.8 (49)			
16 to 20 years	4.6 (23)			
More than 20 years	2.4 (12)			
Number days viewing CSAM in typical month				
Not at all	7.0 (35)	0.02	-0.03	0.004
Several days	54.2 (271)			
More than half the days	21.0 (105)			
Nearly every day	17.8 (89)			
High number of still images viewed per month	16.8 (84)	0.08	0.04	-0.03
High number of videos viewed per month	11.6 (58)	0.10*	0.04	-0.04
Any live stream videos viewed per month	29.0 (145)	0.001	0.01	-0.06
Control over work assigned to you				
No control	26.9 (134)	-0.19***	-0.16***	0.19***
Some control	52.3 (261)			
A lot of control	20.8 (104)			
Frequency of knowing final case resolution				
Never	3.4 (17)	-0.13**	-0.16***	0.20***
Sometimes	31.4 (157)			
Often	35.2 (176)			
All of the time	30.0 (150)			
Typical month frequency of viewing that CSAM that includes… (% often/all the time)				
Children aged 5 or younger	49.6 (248)	0.01	0.003	0.03
Children aged 6 to 10	63.4 (317)	0.03	0.03	0.02
Graphic	72.6 (363)	0.005	0.05	0.01
Sexual contact between a child and adult	65.6 (328)	0.02	0.04	0.03
Penetration of child, including oral sex	64.2 (321)	0.01	0.02	0.05
Violence, beyond the sexual assault	22.2 (111)	-0.04	0.09*	-0.0003
Children posed	61.4 (307)	0.02	0.04	0.03
Multiple children at the same time	33.2 (166)	-0.001	0.05	0.04
Children clearly under influence of alcohol or drugs	8.0 (40)	-0.002	0.07	-0.03
Multiple offenders	23.6 (118)	-0.001	0.06	0.02
Fetishes	31.4 (157)	0.01	0.06	-0.02
Sound	49.2 (246)	0.01	0.05	-0.04

** p ≤ .01; *** p ≤ .001

16.8% of participants had been working CSAM crimes for more than 10 years, 18.6% for 7 to 10 years, 19.4% for 4 to 6 years while many were newer to these types of investigations (45.2% had been working CSAM crimes for 3 years or less). 17.8% of participants viewed CSAM nearly every day in a typical month as part of their job with 21.0% saying they viewed it more than half of the days, 54.2% several days, and 7.0% rarely viewed such material. The number of CSAM still images and recorded videos varied with 16.8% participants reporting they viewed a high number of still images (10,000 or more) and 11.6% a high number of recorded videos (5,000 or more) in a typical month. 29.0% said they viewed any live stream CSAM videos in a typical month. Content viewed in a typical month “often” or “all the time” was extreme – involving young children (63.4% aged 6 to 10 and 49.6% aged 5 or younger), penetration of a child (64.2%), sexual contact between a child and adult (65.6%), and children posed (61.4%). Although not as commonly reported, many participants viewed content that involved violence beyond the sexual assault (22.2%), multiple children at the same time (33.2%), involving multiple offenders (23.6%), fetishes (31.4%), and children clearly under the influence of alcohol or drugs (8.0%). Almost half (49.2%) of participants often viewed CSAM that included sound. Often viewing violent CSAM was significantly related to elevated PTSS (β = 0.09, p ≤ .05). All of the other CSAM content exposures were not significantly related to self-reported mental health, PTSS, or well-being.

Participants were also asked how much control they had over the CSAM work they were assigned and how often they knew of the final case resolution (Table 2). Responses varied – 20.8% said they had a lot of control over the CSAM work they were assigned, 52.3% had some control, and 26.9% had no control. More control was significantly related to less mental health symptoms (β = -0.19, p ≤ .001), lower PTSS scores (β = -0.16, p ≤ .001), and higher well-being scores (β = 0.19, p ≤ .001). 30.0% of participants said they knew the final case resolution all of the time, 35.2% often, 31.4% sometimes, and 3.4% never. More often knowing the final case resolution was significantly related to less mental health symptoms (β = -0.13, p ≤ .01), lower PTSS scores (β = -0.16, p ≤ .001) and higher well-being scores (β = 0.20, p ≤ .001).

### Bivariate relationships between department policies and practices specific to CSAM and mental health

Several departmental policies and practices were related to less mental health symptoms and higher well-being scores at the bivariate level (See Table 3).

**Table 3:** Bivariate relationships between department policies and practices specific to CSAM and mental health

Characteristic	All (n = 500)	Depression/ anxiety β	PTSS β	Well-being β
Preparation before CSAM exposure	29.2 (146)	-0.09*	-0.07	0.11*
Mental health testing/screening prior	6.4 (32)	-0.07	-0.08	0.06
Attended training program related to CSAM	66.6 (333)	0.01	0.02	0.13**
Policies and resources available (CSAM focus)				
Staff meetings where reactions to work-related CSAM is discussed	11.4 (57)	-0.12**	-0.16***	0.15***
Group or individual sessions with mental health professional	21.0 (105)	-0.05	-0.09*	0.12**
Individual case reviews	11.0 (55)	-0.11**	-0.11**	0.13**
Rotations or time limits	4.2 (21)	0.004	0.01	-0.06
Part-time assignments	16.8 (84)	-0.02	-0.01	0.04
Exit interviews or debriefings	4.8 (24)	-0.05	0.001	0.02
Follow-up contact	1.8 (9)	-0.05	-0.09	0.03
Triage person	11.6 (58)	-0.04	-0.04	0.10*
Respect given to CSAM personnel				
None or very little	20.9 (97)	-0.19***	-0.23***	0.20***
Some	49.5 (230)			
A lot	29.7 (138)			
Not sure	7.0 (35)			
Officer wellness program	62.0 (310)	-0.21***	-0.14***	0.17***
Agency…				
Offers ample vacation/personal time off	55.8 (279)	-0.15***	-0.14***	0.22***
Provides daily opportunities for CSAM investigators to debrief	19.6 (98)	-0.16***	-0.18***	0.16***
Provides regular admin updates about positive outcomes from CSAM cases	17.2 (86)	-0.16***	-0.10*	0.15***
Allow you to tend to personal obligations during work hours using own time	58.8 (294)	-0.03	-0.04	0.11*

* p < .05. ** p ≤ .01; *** p ≤ .001.

Having some preparation prior to CSAM exposure (reported by 29.2% of participants) was related to less mental health symptoms and higher well-being scores; having attended a training program related to CSAM (66.6%) was related to higher well-being. Specific policies and practices available in the agency with a CSAM focus were related to less mental health symptoms and greater well-being, including holding staff meetings where reactions to work-related CSAM is discussed (11.4%), group or individual sessions with mental health professionals available (21.0%), and individual case reviews (11.0%). The more respect the participants perceived was given to CSAM personnel in the agency, the less the mental health symptoms and higher the well-being score. Reports varied with 29.7% saying their agency gave a lot of respect to CSAM personnel, 49.5% some respect, and 20.9% none or very little respect.

Participants working in agencies that had an Officer Wellness Program also reported lower mental health symptoms and higher well-being scores. Several opportunities offered by agencies were related to lower mental health scores and higher well-being including offering ample vacation/personal time off (55.8% of participants endorsed this); providing daily opportunities for CSAM investigators to debrief (19.6%); and providing regular administrative updates about positive outcomes from CSAM cases (17.2%).

### Harm mitigation strategies used by forensic examiners and investigators when reviewing CSAM

Investigators reported a variety of different strategies they used while reviewing CSAM to help them cope with the material they were viewing. The percentage of participants who reported they used the strategy “often” varied by the type of strategy (See Table 4).

**Table 4:** Harm mitigation strategies and their bivariate relationships with mental health and well-being

	All (n = 500) % often (n)	Depression/ anxiety β	PTSS β	Well-being β
Strategy				
Take breaks from the material I am reviewing	51.8 (258)	-0.04	-0.09*	0.17***
Listen to music while working	37.4 (186)	0.01	0.002	0.09*
Mask some of the images	6.3 (31)	0.02	0.04	0.06
Turn my mind to pleasant thoughts	27.3 (135)	-0.08	-0.02	0.18***
Talk with someone about what I am viewing	15.9 (79)	-0.11**	-0.09	0.22***
Remind myself of the importance of my work	58.4 (291)	-0.06	0.03	0.15***
Take breaks to meditate or clear my mind	31.9 (159)	-0.06	-0.07	0.18***
Imagine the successful outcome of the case	39.6 (197)	-0.10*	-0.06	0.15***
Take deep breaths	22.9 (114)	0.02	0.04	0.09
Have an alcoholic drink after work	12.1 (60)	0.08	0.15***	-0.16***
Talk to the other officers investigating the case	37.3 (185)	-0.13**	-0.11*	0.18***
Focus my attention on the task	63.3 (315)	-0.06	-0.06	0.18***
Tell myself to try to ignore the harm	16.0 (79)	0.09*	0.11**	-0.05
Get more information about the case	41.5 (206)	-0.02	-0.03	0.08
Try not to focus on any image or video for too long	58.7 (291)	0.005	-0.03	0.10*
Eat sugary snacks or drinks	15.3 (76)	0.21***	0.19***	-0.14***
Reduce the size of the image	11.5 (57)	0.08	0.12**	-0.02
Change the image to black and white	0.4 (2)	-0.02	-0.01	-0.05
Focus on the factors and not the activity in the image	49.3 (245)	-0.11*	-0.02	0.14**
Turn audio down or off	52.7 (262)	-0.01	-0.01	0.12**

* p < .05. ** p ≤ .01; *** p ≤ .001

Strategies commonly used included focusing attention on the task (63.3%), trying not to focus on any image or video for too long (58.7%), reminding myself of the importance of my work (58.4%), turning the audio down or off (52.7%), taking breaks from the material I am viewing (51.8%), focusing on the factors and not the activity in the image (49.3%), and getting more information about the case (41.5%). Other less commonly endorsed strategies are detailed in Table 4.

Strategies related to lower mental health symptoms included talking with someone about what I am doing (β = -0.11, p ≤ .01), imagining the successful outcome of the case (β = -0.10, p ≤ .05), talking with other officers investigating the case (β = -0.13, p ≤ .01), and focusing on the factors and not the activity in the image (β = -0.11, p ≤ .05). Telling myself to try to ignore the harm (β = 0.09, p ≤ .05) and eating sugary snacks or drinks while viewing the material (β = 0.21, p ≤ .001) were related to more mental health symptoms. Lower PTSS scores were found among participants who took breaks form the material they were viewing (β = -0.09, p ≤ .05) and who talked with other officers investigating the case (β = -0.11, p ≤ .05). Those who had an alcohol drink after work (β = 0.15, p ≤ .001), told themselves to try to ignore the harm (β = 0.11, p ≤ .01), ate sugary snacks or drinks (β = 0.19, p ≤ .001), and reduced the size of the image (β = 0.12, p ≤ .01) had higher PTSS scores. Almost all of the individual harm reduction strategies were related to higher well-being scores with the exception of having an alcoholic drink after work and eating sugary snacks or drinks while viewing the CSAM, which were significantly related to less well-being.

### Personal, investigative, and agency factors associated with mental health and well-being

At the multivariate level (See Table 5), having more control over the work assigned to you (β = -0.10, p = .02) and working in an agency with an Officer Wellness Program (β = -0.16, p < .001) were significantly related to lower mental health symptoms in the context of all other characteristics significant at the bivariate level. Eating sugary snacks or drinks while viewing CSAM (β = 0.15, p < .001) was significantly related to more mental health symptoms.

**Table 5:** Multivariate relationships between personal characteristics, agency characteristics, and harm reduction strategies with mental health and well-being

Construct	Depression/ anxiety		PTSS		Well-being	
	β	P value	β	P value	β	P value
CSAM work						
Control over work assigned to you	-0.10	0.02	-0.06	0.21	0.10	0.02
Frequency of knowing final case resolution	-0.06	0.16	-0.09	0.04	0.09	0.04
Preparation before CSAM exposure	-0.05	0.28	---	---	0.04	0.38
Attended training program related to CSAM	---	---	---	---	0.07	0.12
Exposure to CSAM that includes violence	---	---	0.10	0.02	---	---
Agency characteristics						
Staff meetings where reactions to work-related CSAM is discussed	0.02	0.70	-0.05	0.28	0.01	0.74
Group or individual sessions with mental health professional	---	---	-0.005	0.92	0.02	0.70
Individual case reviews	-0.04	0.39	-0.003	0.94	0.06	0.21
Amount of respect given to CSAM personnel	-0.07	0.13	-0.12	0.01	0.09	0.05
Officer Wellness Program	-0.16	< 0.001	-0.09	0.04	0.09	0.04
Agency offers ample vacation/personal time off	-0.02	0.62	-0.02	0.62	0.10	0.03
Agency provides daily opportunities for CSAM investigators to debrief	0.001	0.98	-0.09	0.07	− 0.05	0.29
Agency provides regular admin updates about positive outcomes from CSAM cases	-0.06	0.17	0.03	0.57	0.04	0.42
Agency allow you to tend to personal obligations during work hours using own time	---	---	---	---	0.02	0.68
CSAM harm reduction strategies						
Take breaks from the material I am reviewing	---	---	-0.09	0.05	− 0.03	0.54
Listen to music while working	---	---	---	---	0.07	0.12
Turn my mind to pleasant thoughts	---	---	---	---	0.09	0.08
Talk with someone about what I am viewing	-0.07	0.14	---	---	0.13	0.009
Remind myself of the importance of my work	---	---	---	---	0.01	0.77
Take breaks to meditate or clear my mind	---	---	---	---	0.09	0.07
Imagine the successful outcome of the case	0.04	0.40	---	---	0.02	0.71
Have an alcoholic drink after work ^a^	---	---	0.13	0.002	− 0.17	< 0.001
Talk to the other officers investigating the case	-0.02	0.73	-0.03	0.56	0.004	0.93
Focus my attention on the task	---	---	---	---	0.14	0.002
Tell myself to try to ignore the harm	0.06	0.17	0.05	0.21	---	---
Try not to focus on any image or video for too long	**---**	---	---	---	− 0.06	0.26
Eat sugary snacks or drinks	0.15	< 0.001	0.12	0.01	− 0.09	0.03
Reduce the size of the image	---	---	0.10	0.02	---	---
Focus on the factors and not the activity in the image	-0.08	0.08	---	---	0.03	0.61
Turn audio down or off	---	---	---	---	0.08	0.11
Demographic characteristics						
Female	0.01	0.73	-0.03	0.45	0.07	0.11
Have children or grandchildren	0.06	0.17	-0.02	0.68	− 0.04	0.41
White race	0.05	0.23	-0.04	0.36	− 0.04	0.35
Married	-0.08	0.10	-0.05	0.28	0.15	0.002
Number of years in police work	-0.02	0.63	-0.02	0.64	0.04	0.40

Lower PTSS scores were found for investigators who more frequently knew the final resolution of the case (β = -0.09, p = .04), worked in agencies where more respect was given to CSAM personnel (β = -0.12, p = .01), had an Officer Wellness Program (β = -0.09, p = .04), and took breaks from the material being viewed (β = -0.09, p = .05). Higher PTSS scores were found for those who were more often exposed to violent CSAM (β = 0.10, p = .02), had an alcoholic drink after work (β = 0.13, p = .002), ate sugary snacks or drinks when reviewing material (β = 0.12, p = .01), and reduced the size of the image (β = 0.10, p = .02), holding all other factors constant.

Factors significantly related to higher well-being scores were mostly consistent with what was found with mental health; having more control over work assigned to you (β = 0.10, p = .02), more frequently knowing the final case resolution (β = 0.09, p = .04), and working in an agency that has an Officer Wellness Program (β = 0.09, p = .04). Working in an agency where more respect is given to CSAM personnel (β = 0.09, p < .05) and that offers ample vacation/personal time off (β = 0.10, p = .03) was related to better well-being. After considering each harm mitigation strategy significant at the bivariate level, talking with someone about what they were viewing (β = 0.13, p = .009) and focusing attention on the task (β = 0.14, p = .002) were related to higher well-being scores, while having an alcohol drink after work (β = -0.17, p < .001) and eating sugary snacks or drinks (β = -0.09, p = .03) were related to lower well-being scores. Being married was related to higher well-being scores.

## Discussion

Although CSAM investigators and forensic examiners are frequently exposed to a great amount of extreme content as part of their professions, findings from the current study indicate that the frequency of exposure was not systematically related to mental health conditions. This finding aligns with a previous study in which investigators of CSAM asserted that viewing disturbing content was not the most difficult part of their job, but instead described organizational factors as having much more impact on their stress levels [31]. Nonetheless, as suggested by prior qualitative research [35], our findings identify that there is substantial variation in the degree of stress and well-being among police officers who review CSAM, and that this is influenced to some degree by the types and content of material, the viewing context, and individual, case-related, and organizational factors. For example, our findings indicated that frequent exposure to CSAM with violent content was related to higher PTSS.

While the extent of CSAM exposure was not related to mental health symptoms, findings indicate that agency-level factors were related to mental health and well-being among CSAM investigators. This finding is encouraging as these are organizational factors that may be easy to implement in a low cost and low resource manner: for example, providing investigators more control over the work that is assigned to them and taking the time to keep them apprised of final case resolutions. Additional agency policies and practices that were associated with lower levels of mental health and increased well-being included daily opportunities for CSAM investigators to debrief, and provision of regular updates about positive outcomes from CSAM cases. These agency-level factors likely help foster a departmental culture that is supportive of the intense and perhaps unique work CSAM investigators undertake and thereby contribute to the mental health and well-being of law enforcement. Indeed, investigators and forensic examiners who worked in agencies where more respect was given to this type of work reported higher well-being and lower mental health symptoms in the current study.

Officer Wellness Programs also appeared to be an important resource for helping ensure the well-being of CSAM investigators and improving mental health at the agency level. These programs are not universal, existing in only 60% of agencies that conducted CSAM investigations in one study [23]. Overall, our findings correspond with previous studies indicating agency-level practices and policies as well as attitudes towards police work to be important factors that can help mitigate stress – or amplify it. Exacerbating conditions noted in previous research include intra-agency conflicts and tensions [22, 31], such as decisions made without consultation, conflict over how to perform tasks, and lack of recognition and appreciation from commanders [31].

It appears CSAM investigators and forensic examiners may have different resilience strategies. The resilience of CSAM investigators was demonstrated in this study by the different strategies they implemented while viewing CSAM which appeared to help them reduce the potential for harm. Strategies that promoted partnership and collaboration (e.g., talking with other investigators or someone about what they were viewing) appeared to be particularly beneficial for mental health. A substantial number of additional strategies were related to well-being: focusing on other features than the activity in the image, turning audio down or off, trying not to stay on any image or video for too long, keeping attention on the task, entertaining pleasant thoughts, reminding oneself of the importance of their work, taking breaks, and listening to music while working. These more detailed findings add to previous work indicating frequent use of positive coping mechanisms is associated with increased satisfaction from helping others (compassion satisfaction), decreased symptoms of posttraumatic stress and burnout [36], and have the potential to mitigate the potential harm of the job [25]. Many of these strategies would be relatively easy to implement as part of CSAM investigation training programs.

Other coping strategies appeared to be associated with higher mental health symptoms and reduced well-being– for example, having an alcoholic drink after work and eating sugary snacks or drinks while viewing the material. These behaviors could be indicative of a broader unhealthy lifestyle with more global mental and/or physical health conditions that need to be addressed. Turning to maladaptive coping mechanisms such as alcohol use may occur when investigators are unable to successfully process their emotional pain and reactions to CSAM [37]. Thus, it is important to identify the coping strategies that mitigate the negative consequences of viewing CSAM and encourage their use.

### Limitations

The current study had a few limitations that should be noted when considering the implications of the findings. First, data were collected via a convenience sample, which might not be representative of the population of investigators/forensic examiners who view CSAM. Moreover, it is possible, that there was a built-in bias to a study on police wellness, in which law enforcement who are more resilient – or more troubled - were the ones more willing to complete a survey on their CSAM exposure and mental health and well-being. Second, the study was based on self-report measures, which are subject to response bias (e.g., under- or over-reporting). Police may have particular biases against acknowledging mental health symptoms [38]. Third, the design was cross-sectional; therefore, causal relations between study variables cannot be inferred.

### Future research

Our findings suggest that the extent of investigator/examiner exposure to CSAM was unrelated to mental health conditions or well-being – in terms of duration of time working these cases, frequency of viewing CSAM per month, amount of CSAM viewed, and the content itself (apart from violent CSAM, which was related to PTSS). Although the current study supports the idea that agency-level factors and personal strategies might buffer against the effect of CSAM exposure, additional moderating variables should be examined in future research (e.g., social support, cognitive schemas, emotional regulation). Our study also indicated some maladaptive coping strategies (e.g., having an alcoholic drink after work) were related to elevated mental health conditions and lower well-being. A more in-depth investigation is needed focusing on substance use in CSAM investigators, as it is unclear how often substances are used for regulating emotional pain or as a distraction. In addition, the current study only examined depression, anxiety and PTSS; future research could include additional mental health conditions such as somatization, suicidal thoughts, and dissociation. Moreover, as Officer Wellness Programs were strongly related to investigators’ mental health and well-being, future studies might shed light on this topic by examining the characteristics and content of these programs. Finally, social media platforms are increasingly turning the search and removal of contraband images over to civilian content monitors [39] who may not have some of the resilience factors displayed by law enforcement employees. This research needs to be extended to these workers as well.

## Conclusions

Findings from this paper support the idea that where there is stress, there is resilience. Although CSAM investigators have stressful work in which they are frequently exposed to a great amount of extreme content, such exposure is, for the most part, not related to mental health conditions among these investigators. Different agency-level factors and personal strategies used to regulate the stress associated with the work are related to lower levels of mental health conditions and increased well-being.

## Data Availability

The dataset used during the current study is available from the corresponding author on reasonable request.
